# Efficacy of bacterial ribosomal RNA-targeted reverse transcription-quantitative PCR for detecting neonatal sepsis: a case control study

**DOI:** 10.1186/1471-2431-10-53

**Published:** 2010-07-29

**Authors:** Makoto Fujimori, Ken Hisata, Satoru Nagata, Nobuaki Matsunaga, Mitsutaka Komatsu, Hiromichi Shoji, Hiroaki Sato, Yuichiro Yamashiro, Takashi Asahara, Koji Nomoto, Toshiaki Shimizu

**Affiliations:** 1 Department of Pediatrics, Juntendo University School of Medicine, Tokyo, Japan; 2 Division of Laboratory for Probiotics Research, Juntendo University, Tokyo, Japan; 3 Yakult Central Institute for Microbiological Research, Tokyo, Japan

## Abstract

**Background:**

Neonatal sepsis is difficult to diagnose and pathogens cannot be detected from blood cultures in many cases. Development of a rapid and accurate method for detecting pathogens is thus essential. The main purpose of this study was to identify etiological agents in clinically diagnosed neonatal sepsis using bacterial ribosomal RNA-targeted reverse transcription-quantitative PCR (BrRNA-RT-qPCR) and to conduct comparisons with the results of conventional blood culture. Since BrRNA-RT-qPCR targets bacterial ribosomal RNA, detection rates using this approach may exceed those using conventional PCR.

**Methods:**

Subjects comprised 36 patients with 39 episodes of suspected neonatal sepsis who underwent BrRNA-RT-qPCR and conventional blood culture to diagnose sepsis. Blood samples were collected aseptically for BrRNA-RT-qPCR and blood culture at the time of initial sepsis evaluation by arterial puncture. BrRNA-RT-qPCR and blood culture were undertaken using identical blood samples, and BrRNA-RT-qPCR was performed using 12 primer sets.

**Results:**

Positive rate was significantly higher for BrRNA-RT-qPCR (15/39, 38.5%) than for blood culture (6/39, 15.4%; p = 0.0039). BrRNA-RT-qPCR was able to identify all pathogens detected by blood culture. Furthermore, this method detected pathogens from neonates with clinical sepsis in whom pathogens was not detected by culture methods.

**Conclusions:**

This RT-PCR technique is useful for sensitive detection of pathogens causing neonatal sepsis, even in cases with negative results by blood culture.

## Background

The incidence of sepsis is higher in neonates than in adult patients, and the risk of mortality is higher [1]. In particular, neonates with low birth weight show relatively high morbidity and mortality [2-4]. Furthermore, neonatal sepsis is difficult to diagnose, as clinical signs are often obscure and laboratory parameters are unspecific. No clear-cut definition of neonatal sepsis has been agreed to, although several studies have tried to identify one [5-7]. Clinicians thus permit over-treatment based on the high risk of mortality if sepsis is left untreated. Normally, when the clinician suspects neonatal infection or sepsis, blood culture and cultures of various body sites are immediately undertaken and administration of broad-spectrum antimicrobial agents is empirically started. Although blood cultures are usually the basis for a diagnosis of sepsis due to bloodstream infection, culture results are reported after several days and sensitivity is particularly low for neonates [8,9]. We have encountered many cases in which pathogens have not been detected from culture even though sepsis was clinically suspected. Antimicrobial agents thus could not be de-escalated, necessitating continued administration of broad-spectrum antimicrobial agents. However, over-administration of broad-spectrum antimicrobial agents is associated with increased development of drug-resistant microorganisms and disturbance of intestinal flora [3,4]. In addition, prolonged initial empirical antimicrobial therapy may be associated with increased risk of necrotizing enterocolitis or death among extremely low birth weight neonates [2]. For these reasons, development of pathogen-detection tools assisting blood culture that offer more rapid results and higher sensitivity is expected in neonatal intensive care to optimize the use of antimicrobial agents.

PCR techniques have been used for the detection of microorganisms from neonates with suspected sepsis instead of blood culture [10]. PCR techniques are becoming more useful in decreasing laboratory turnaround times, providing results to the clinician at an earlier stage. Several reports have described the use of PCR-based assays for rapid and accurate identification of bacterial DNA in the blood of neonates with suspected or confirmed sepsis [1,4,11-17]. These assays rely on PCR amplification of the 16S rRNA gene, a highly conserved gene present in all bacterial species but absent in humans. Although molecular assays may improve the detection of pathogens causing sepsis, the positivity rate for PCR in various studies of septic neonates remains low [10].

The recently developed method of bacterial ribosomal RNA-targeted reverse transcription-quantitative PCR (BrRNA-RT-qPCR) assay has the potential to overcome many of these problems. Matsuda et al. [18] reported that RT-qPCR assay requires an average of only 6 h for the quantification of various kinds of commensal bacteria. In addition, this approach is sensitive enough to detect and quantify bacteria present at < 10 CFU/ml of blood by targeting bacterial ribosomal RNA.

The purpose of the present study was to identify etiological agents in clinically diagnosed neonatal sepsis using bacterial ribosomal RNA-targeted reverse transcription-quantitative PCR (BrRNA-RT-qPCR) and conduct comparisons with the results of conventional blood culture. This investigation also evaluated whether the qPCR test could detect rRNA of organisms in culture-negative patients with neonatal sepsis.

## Methods

### Patients

The study was approved by the Clinical Research Ethics Committee of Juntendo University Hospital and written parental consent was obtained before enrolment of each subject in the study. All included neonates were admitted to the neonatal intensive care units (NICUs) at Juntendo University Hospital or Juntendo University Shizuoka Hospital between February and August 2009.

### Neonatal sepsis criteria

The attending physician in each case suspected neonatal sepsis based on a rise in C-reactive protein (CRP) and clinical assessment of signs including fever, temperature instability, apnea, dyspnea, cyanosis, respiratory distress, vomiting, abdominal distension, not-doing-well, irritability, poor feeding, and hyperglycemia. Neonates who met two or more of these criteria were enrolled in the study.

### Clinical sepsis criteria

Various neonatal sepsis criteria have been investigated in previous reports, but have shown low sensitivity and specificity. Therefore, no clear, accepted criteria for neonatal sepsis currently exist. The possibility that cases of non-infection, local infection, viral infection and so on might be enrolled by broad interpretation of criteria in a judgment only assessing onset is thus a matter of concern. Consequently, clinical sepsis (CSEP) was diagnosed in a retrospective manner by two neonatologists after each neonate was discharged from the NICU, based on clinical course such as: two or more signs of infection; organ dysfunction (respiratory failure, pulmonary hypertension, cardiac failure, shock, renal failure, liver dysfunction, cerebral edema or thrombosis, adrenal hemorrhage and/or insufficiency, bone marrow dysfunction and disseminated intravascular coagulopathy); laboratory data; other cultures (urine, cerebrospinal fluid and cord blood) if clinically indicated; and therapeutic response.

### Sample collection and study design

In total, 1.5-3 ml of whole blood was collected aseptically by arterial puncture at the time of initial sepsis evaluation. Each sample was divided into three vials: an RNA protective tube; an aerobic blood culture bottle; and an anaerobic blood culture bottle. In most cases, two blood samples from different body sites were taken to avoid confusion caused by skin contamination, which was likely to represent the leading cause of false-positive results from this PCR method and to increase the likelihood of bacterial detection [19].

### Blood cultures

Each blood culture sample was subdivided into a two-bottle set (92F aerobic and 93F anaerobic resin blood culture bottles; Becton Dickinson, Tokyo, Japan), and processed in an automatic culture device (Bactec 9240; Becton Dickinson) within 1 h after collection at the institutional laboratory of Juntendo University Hospital or Juntendo University Shizuoka Hospital.

### RNA preparation

Each blood sample was mixed with 2 ml of RNAprotect™ Bacterial Reagent (Qiagen, Hilden, Germany), and then was set to rest at room temperature for 5 min. The mixture was stored at -80°C until analysis. These samples were thawed at room temperature and centrifuged at 12,000 × *g *and 4°C for 10 min and the supernatant was carefully removed. The sample was resuspended in a solution containing 346.5 μl RLT lysis buffer (Qiagen Sciences, Germantown, MD), 3.5 μl of β-mercaptoethanol (Sigma-Aldrich, St. Louis, MO), and 100 μl of Tris-ethylenediaminetetraacetic acid (EDTA) buffer (pH 8.0). Next, 300 mg of glass beads (diameter, 0.1 mm; BioSpec Products, Bartlesville, OK) was added to the suspension and processed in a high-speed homogenizer (Shake Master, Bio Medical Science, Tokyo, Japan) for 5 min to achieve cell lysis. Next, 500 μl of acid phenol (Wako Pure Chemical Industries, Osaka, Japan) was added. After incubation for 10 min at 60°C, 100 μl of chloroform-isoamyl alcohol was added to the mixture and centrifuged at 12,000 × *g *and 4°C for 5 min. In the next step, 450 μl of supernatant was collected and added to an equal volume of chloroform-isoamyl alcohol. After centrifugation at 12,000 × *g *and 4°C for 5 min, 400 μl of the supernatant was collected. After the addition of 400 μl of isopropanol and 40 μl of 3 M sodium acetate, the mixture was centrifuged at 15,000 × *g *at 4°C for 5 min. The supernatant was removed and 500 μl of 80% ethanol was added to the pellet. After centrifugation at 15,000 × *g *and 4°C for 2 min, the supernatant was removed and the pellet was dried. Finally, the nucleic acid fraction was suspended in 100 μl of nuclease-free water.

### Primers

Numerous 16S rRNA gene sequences have been described previously. Sequences used in the present study were as follows: *Streptococcus agalactiae *forward: s-Sag-F, reverse: g-Sag-R [20]; genus *Staphylococcus *forward: STPYF, reverse: STPYR2 [21]; genus *Enterococcus *forward: g-Encoc-F, reverse: g-Encoc-R [18]; family *Enterobacteriaceae *forward: En-lsu-3F, reverse: En-lsu-3R [18]; genus *Pseudomonas *forward: PSD7F, reverse: PSD7R [18]; *Bacillus cereus *forward: S-S-Bc-200-a.S-18 [22], reverse: Bc2R [22,23]; *Bacteroides fragilis *group forward: g-Bfra-F2, reverse: g-Bfra-R [24]; *Atopobium *cluster forward: c-Atopo-F, reverse: c-Atopo-R [24]; *Clostridium coccoides *group forward: g-Ccoc-F, reverse: g-Ccoc-R [24]; *Clostridium leptum *subgroup forward: sg-Clept-F, reverse: sg-Clept-R3 [24]; *Clostridium perfringens *forward: s-Clper-F, reverse: ClPER-R [24]; genus *Bifidobacterium *forward: g-Bifid-F, reverse: g-Bifid-R [24]; and genus *Prevotella *forward: g-Prevo-F, reverse: g-Prevo-R [24].

### RT-qPCR

The RT-qPCR assay was performed using the methods described previously by Matsuda *et al*. [18]. The RT-qPCR assay was performed using a One Step RT-PCR kit (Qiagen). Amplification reactions were performed in a volume of 20 μl containing 2 μl of template RNA, 1 × OneStep RT-PCR Buffer (Qiagen), deoxynucleoside triphosphate at a concentration of 0.5 mM, 1:100,000 dilution of SYBR green I (BioWhittaker Molecular Applications, Rockland, ME), 0.8 μl of OneStep RT-PCR Enzyme mixture (Qiagen) and each of the specific primers at a concentration of 0.75 μM. The reaction mixture was incubated at 50°C for 30 min for reverse transcription. The continuous amplification program consisted of one cycle at 95°C for 15 min, followed by 40 cycles at 94°C for 20 s, 60°C (*Staphylococcus, Enterobacteriaceae, Pseudomonas, Bacillus cereus, S. agalactiae*), 55°C (*Enterococcus, Clostridium coccoides *group, *Clostridium leptum, Atopobium *cluster, *Prevotella, Bifidobacterium *) or 50°C (*Bacteroides fragilis *group) for 20 s and 72°C for 50 s. Fluorescent products were detected in the last step of each cycle. Both amplification and detection were performed using an ABI PRISM 7500HT sequence detection system (Applied Biosystems, Foster City, CA).

Determination of the number of bacteria in a sample was performed essentially as described previously [18,24]. A calibration curve was constructed by establishing a threshold line and plotting the threshold cycle (Ct) value, representing the cycle number at which threshold fluorescence was reached, and then the corresponding cell count was determined using a Sequence Detection System (Applied Biosystems). The entire BrRNA-RT-qPCR assay, including the RNA extraction step, could be completed in about 6 h.

### Statistical analysis

Results were analyzed using SAS version 8.02 software (CISCO, Tokyo, Japan). Quantitative data are presented as mean ± standard deviation. McNemar's test with continuity correction was performed to analyze associations between the results of BrRNA-RT-qPCR and blood culture. Two-tailed values of *P *< 0.05 were considered statistically significant.

## Results

### Participants

Individual patient characteristics are summarized in Additional file 1 . A total of 65 samples were obtained from 36 patients throughout the 39 episodes of neonatal sepsis in this study. Double samples from different body sites were able to be taken from 24 patients with 26 septic episodes, while only a single sample was obtained from 12 cases with 13 episodes due to technical problems. Moreover, we excluded repeated samples taken in the same episode.

### Result of BrRNA-RT-qPCR compared to blood culture in total patients

The positive rate was significantly higher for BrRNA-RT-qPCR (15/39, 38.5%) than for blood culture (6/39, 15.4%; p = 0.0039) (Additional file 2). Types and amount of pathogens detected by BrRNA-RT-qPCR are shown in Additional file 3. Blood culture detected pathogens in six of 39 episodes, identifying *Staphylococcus epidermidis *(n = 2), *Klebsiella pneumonia *(n = 3), *Enterobacter cloacae *(n = 1) and *Pseudomonas aeruginosa *(n = 1). BrRNA-RT-qPCR could detect pathogens in 15 of 39 episodes, identifying *Staphylococcus *(n = 2), *Enterobacteriaceae *(n = 11), *Pseudomonas *(n = 1) and *Clostridium leptum *subgroup (n = 1) in the PCR-positive group. In all six blood culture-positive episodes, BrRNA-RT-qPCR detected the same type of bacteria as that identified by the culture. Polymicrobial infection was identified by both PCR and blood culture in one episode (Case 27). In 9 of the 15 episodes in which pathogens were detected by BrRNA-RT-qPCR, culture method could not detect pathogens from blood.

### Result of BrRNA-RT-qPCR in comparison to blood culture in patients with blood samples obtained from two sites and clinical diagnosis

Blood samples were obtained from two sites in 26 episodes with suspected neonatal sepsis. The positive rate was significantly higher for BrRNA-RT-qPCR (10/26, 38.5%) than for blood culture (3/26, 11.5%; p = 0.0156). All three culture-positive/PCR-positive episodes showed clinical sepsis. Seven of the 26 episodes that were positive according to PCR, but showed negative results from blood culture are presented in Additional file 4. Among those, four cases (Cases 4, 5, 7 and 9) clearly displayed clinical sepsis and disseminated intravascular coagulation (DIC) (Additional file 4). In Case 4, > 10^5 ^CFU/mL of *E. coli *was detected in urine collected through a catheter, and presence of *Enterobacteriaceae *consistent with *E. coli *was detected according to the results of the present PCR method. In Case 7, *E. coli *was identified from maternal umbilical blood culture, and the present PCR method of a blood sample from the neonate detected *Enterobacteriaceae *consistent with *E. coli*. Cases 5 and 9 were severe cases of sepsis with DIC, but blood cultures were negative. The other 16 episodes showed culture-negative/PCR-negative results, but three of these episodes were diagnosed with clinical sepsis.

## Discussion

Reasons for lower sensitivity of blood culture are related to the low numbers of bacteria within the bloodstream, the small volumes of blood obtained from neonates for culture, and the increasingly common practice of providing intrapartum antibiotic prophylaxis to mothers of high-risk deliveries and high-risk neonates [4,14,25,26]. Further efforts toward the development of methods to detect neonatal sepsis are thus focused on improving sensitivity, shortening the identification time for pathogens and improving optimized use of antibiotics. A rapid diagnosis of neonatal sepsis permits earlier administration of appropriate antimicrobial agents and reduces morbidity and mortality. The 16S rDNA sequencing offer the following advantages over blood cultures: 1) use of smaller volumes of blood; 2) production of results within a shorter turnaround time; 3) ability to detect a small amount of bacteria; and 4) reduced likelihood of effects from prior antimicrobial therapy. However, various studies of neonatal sepsis have reported no significant difference between blood culture and PCR targeting rDNA, and the detection rate of PCR techniques reportedly shows a range of 3-29% [1,10,11,13,16,17].

On the other hand, BrRNA-RT-qPCR may represent a more sensitive method by targeting ribosomal RNA. Matsuda et al. [18] had focused on rRNA as the target for precise and sensitive quantification of commensal subdominant bacterial populations, since rRNA is a universal constituent of bacterial ribosomes and high copy numbers (10^3^-10^4 ^molecules per actively growing cell) are present as housekeeping genes. In peripheral blood, the lower detection limit for BrRNA-RT-qPCR quantification of bacteria was 2 cells/ml of blood. Sensitivity was approximately 10- to 100-fold higher than that of conventional PCR methods [18]. In this study, the detection rate using this BrRNA-RT-qPCR assay was much higher than the conventional blood culture in neonatal sepsis. One pathogenic cell per milliliter of culture could be detected using this method. This is the first study in which 16S ribosomal RNA-targeted RT-qPCR was used in neonatal sepsis to detect bacterial pathogens; other studies have performed detection using 16S rRNA gene or rDNA. Moreover, the distribution of pathogens isolated from neonatal sepsis is based on a result of blood cultures with lower sensitivity. In this study, *Enterobacteriaceae *were detected most from blood samples. This indicates the possibility that the frequency of pathogens for neonatal sepsis based on this method differs from results based on blood culture. Enormous variance in antibiotic susceptibility exists between some genera in the family *Enterobacteriaceae*, but clarification of genus information enables suspension of unnecessary antibiotics, such as suspending use of vancomycin for patients showing methicillin-resistant *Staphylococcus aureus*. We are planning to develop primers suitable for discriminating between these genera.

Since no widely accepted definition of neonatal sepsis has been created, the criteria for neonatal sepsis may differ between institutions and clinicians. Detection rates of pathogens from the bloodstream also vary between institutions. We therefore cannot simply compare the sensitivity of this method with previous PCR methods. However, this method showed a detectability about three-times that of conventional blood cultures in this study. Methods offering more sensitivity than this method in comparison with blood culture have not been reported previously. Clinical applications thus appear feasible for this method.

To confirm the clinical efficacy of this method, we retrospectively reviewed the clinical courses of 26 episodes for which two sets of blood samples had been taken. Seven of 26 episodes showed positive detection of pathogens using this method, despite negative blood cultures. In the field of neonatal care, cases that test negative in blood cultures despite clinical suspicion of sepsis have become a problem [5]. If prompt specification of pathogens in such cases becomes possible, the present method would be clinically important and may also facilitate selection of the most appropriate treatment. In particular, molecular techniques are considered more useful than traditional methods for those cases in which the use of antimicrobial agents that make blood cultures prone to negative results has been initiated and for cases with small amounts of pathogen that are difficult to culture and sample [14]. Regarding the remaining three cases (Cases 6, 8 and 10), increased inflammatory reactions were observed in all cases, but obvious infectious signs were observed irritability in Cases 6 and 10. All these cases were initially suspected as maternal and intrauterine infection, and antimicrobial agents had already been administered to the mothers. Furthermore, onset was within 24 h of birth in all cases. Pathogens were considered to have avoided detection and no obvious symptoms were observed due to the administration of antimicrobial agents to the mothers. Three of the other 16 episodes showing culture-negative/PCR-negative results were clinically diagnosed clinical sepsis. Three of 16 episodes with negative results for both blood culture and PCR were actually diagnosed as sepsis. These were identified as false-negative results for the following reasons: 1) microorganisms in the bloodstream are not generally distributed in a uniform manner and may not be captured from only a small blood sample; 2) our PCR primers may not cover all of the pathogens at the present time; and 3) our primers could not cover fungi, which have become a larger problem in neonatal sepsis. Improvements in primers for detecting bacteria and fungi remains a major issue to be addressed in future studies. At present, BrRNA-RT-qPCR is expensive and labor-intensive. In the future, we plan to create a simple and compact system for detecting pathogens in hospital settings.

## Conclusions

As neonatal sepsis has to be diagnosed based on a limited sample volume, it is a very difficult diagnosis to make. Under these circumstances, identification of pathogens within approximately 6 h using only 0.5 ml of blood would facilitate prompt diagnosis and treatment of neonatal sepsis. According to the above results, BrRNA-RT-qPCR appears to provide prompt findings with higher detection sensitivity as compared to blood culturing, indicating the possibility that this method is a useful adjunct to blood culture for diagnosing neonatal sepsis.

## Ccompeting interests

The authors declare that they have no competing interests.

## Authors' contributions

MF, KH, SN, TA, KN and TS were responsible for the study design. MF, TA and KN modified, validated and performed the BrRNA-RT-qPCR analysis. KH, NM, MK, HSh and HS were responsible for the clinical study implementation. MF carried out the data analysis and drafted the manuscript. All authors participated in writing and/or reviewing of this manuscript. All authors read and approved the final manuscript.

## Pre-publication history

The pre-publication history for this paper can be accessed here:

http://www.biomedcentral.com/1471-2431/10/53/prepub

## Supplementary Material

Additional file 1 **Table 1: Baseline characteristics of patients**. Click here for file

Additional file 2 **Table 2: Comparison of BrRNA-RT-qPCR and blood culture**. Click here for file

Additional file 3 **Table 3: Positive results using the BrRNA-RT-qPCR method**. Click here for file

Additional file 4 **Table 4: Summary of patients with positive-BrRNA-RT-qPCR/negative-blood culture in 2 sets of blood samples**. Click here for file

## References

[B1] Jordan JA Durso MB Butchko AR Jones JG Brozanski BS Evaluating the near-term infant for early onset sepsis: progress and challenges to consider with 16S rDNA polymerase chain reaction testing J Mol Diagn 2006 8 3 357 363 10.2353/jmoldx.2006.050138 16825509 PMC1867603

[B2] Cotten CM Taylor S Stoll B Goldberg RN Hansen NI Sanchez PJ Ambalavanan N Benjamin DK Prolonged duration of initial empirical antibiotic treatment is associated with increased rates of necrotizing enterocolitis and death for extremely low birth weight infants Pediatrics 2009 123 1 58 66 10.1542/peds.2007-3423 19117861 PMC2760222

[B3] Del Vecchio A Laforgia N Capasso M Iolascon A Latini G The role of molecular genetics in the pathogenesis and diagnosis of neonatal sepsis Clin Perinatol 2004 31 1 53 67 10.1016/j.clp.2004.03.012 15183656

[B4] Jordan JA Durso MB Real-time polymerase chain reaction for detecting bacterial DNA directly from blood of neonates being evaluated for sepsis J Mol Diagn 2005 7 5 575 581 16258155 10.1016/S1525-1578(10)60590-9 PMC1867550

[B5] Kaufman D Fairchild KD Clinical microbiology of bacterial and fungal sepsis in very-low-birth-weight infants Clin Microbiol Rev 2004 17 3 638 680 table of contents 10.1128/CMR.17.3.638-680.2004 15258097 PMC452555

[B6] Ng PC Lam HS Diagnostic markers for neonatal sepsis Curr Opin Pediatr 2006 18 2 125 131 10.1097/01.mop.0000193293.87022.4c 16601490

[B7] Malik A Hui CP Pennie RA Kirpalani H Beyond the complete blood cell count and C-reactive protein: a systematic review of modern diagnostic tests for neonatal sepsis Arch Pediatr Adolesc Med 2003 157 6 511 516 10.1001/archpedi.157.6.511 12796229

[B8] Chan KY Lam HS Cheung HM Chan AK Li K Fok TF Ng PC Rapid identification and differentiation of Gram-negative and Gram-positive bacterial bloodstream infections by quantitative polymerase chain reaction in preterm infants Crit Care Med 2009 37 8 2441 2447 10.1097/CCM.0b013e3181a554de 19531943

[B9] Lam HS Ng PC Biochemical markers of neonatal sepsis Pathology 2008 40 2 141 148 10.1080/00313020701813735 18203036

[B10] Frayha HH Kalloghlian A Gram-specific quantitative polymerase chain reaction for diagnosis of neonatal sepsis: implications for clinical practice Crit Care Med 2009 37 8 2487 2488 10.1097/CCM.0b013e3181af1bd1 19609126

[B11] Jordan JA Durso MB Comparison of 16S rRNA gene PCR and BACTEC 9240 for detection of neonatal bacteremia J Clin Microbiol 2000 38 7 2574 2578 10878046 10.1128/jcm.38.7.2574-2578.2000 PMC86972

[B12] Laforgia N Coppola B Carbone R Grassi A Mautone A Iolascon A Rapid detection of neonatal sepsis using polymerase chain reaction Acta Paediatr 1997 86 10 1097 1099 10.1111/j.1651-2227.1997.tb14815.x 9350892

[B13] Shang S Chen G Wu Y Du L Zhao Z Rapid diagnosis of bacterial sepsis with PCR amplification and microarray hybridization in 16S rRNA gene Pediatr Res 2005 58 1 143 148 10.1203/01.PDR.0000169580.64191.8B 15985688

[B14] Woo PC Lau SK Teng JL Tse H Yuen KY Then and now: use of 16S rDNA gene sequencing for bacterial identification and discovery of novel bacteria in clinical microbiology laboratories Clin Microbiol Infect 2008 14 10 908 934 10.1111/j.1469-0691.2008.02070.x 18828852

[B15] Ohlin A Backman A Bjorkqvist M Molling P Jurstrand M Schollin J Real-time PCR of the 16S-rRNA gene in the diagnosis of neonatal bacteraemia Acta Paediatr 2008 97 10 1376 1380 10.1111/j.1651-2227.2008.00924.x 18624992

[B16] Reier-Nilsen T Farstad T Nakstad B Lauvrak V Steinbakk M Comparison of broad range 16S rDNA PCR and conventional blood culture for diagnosis of sepsis in the newborn: a case control study BMC Pediatr 2009 9 5 10.1186/1471-2431-9-5 19152691 PMC2635358

[B17] Wu YD Chen LH Wu XJ Shang SQ Lou JT Du LZ Zhao ZY Gram stain-specific-probe-based real-time PCR for diagnosis and discrimination of bacterial neonatal sepsis J Clin Microbiol 2008 46 8 2613 2619 10.1128/JCM.02237-07 18550744 PMC2519463

[B18] Matsuda K Tsuji H Asahara T Kado Y Nomoto K Sensitive quantitative detection of commensal bacteria by rRNA-targeted reverse transcription-PCR Appl Environ Microbiol 2007 73 1 32 39 10.1128/AEM.01224-06 17071791 PMC1797142

[B19] Kellogg JA Manzella JP Bankert DA Frequency of low-level bacteremia in children from birth to fifteen years of age J Clin Microbiol 2000 38 6 2181 2185 10834973 10.1128/jcm.38.6.2181-2185.2000 PMC86758

[B20] Sakaguchi S Saito M Tsuji H Asahara T Takata O Fujimura J Nagata S Nomoto K Shimizu T Bacterial Ribosomal RNA-Targeted Reverse Transcription PCR Used to Identify Pathogens Responsible for Fever with Neutropenia J Clin Microbiol 2010 48 5 1624 1628 10.1128/JCM.01724-09 20351213 PMC2863901

[B21] Wada M Lkhagvadorj E Bian L Wang C Chiba Y Nagata S Shimizu T Yamashiro Y Asahara T Nomoto K Quantitative reverse transcription-PCR assay for the rapid detection of methicillin-resistant Staphylococcus aureus J Appl Microbiol 2010 108 3 779 788 10.1111/j.1365-2672.2009.04476.x 19702857

[B22] Hansen BM Leser TD Hendriksen NB Polymerase chain reaction assay for the detection of Bacillus cereus group cells FEMS Microbiol Lett 2001 202 2 209 213 10.1111/j.1574-6968.2001.tb10805.x 11520616

[B23] Tsujih H Matsuda K Asahara T Kiwaki M Nomoto K Quantitative analysis of microbes with rRNA as target 2006 In., vol. PCT/JP2006/301467. Japan;

[B24] Matsuda K Tsuji H Asahara T Matsumoto K Takada T Nomoto K Establishment of an analytical system for the human fecal microbiota, based on reverse transcription-quantitative PCR targeting of multicopy rRNA molecules Appl Environ Microbiol 2009 75 7 1961 1969 10.1128/AEM.01843-08 19201979 PMC2663197

[B25] Neal PR Kleiman MB Reynolds JK Allen SD Lemons JA Yu PL Volume of blood submitted for culture from neonates J Clin Microbiol 1986 24 3 353 356 3760131 10.1128/jcm.24.3.353-356.1986 PMC268912

[B26] Peters RP van Agtmael MA Danner SA Savelkoul PH Vandenbroucke-Grauls CM New developments in the diagnosis of bloodstream infections Lancet Infect Dis 2004 4 12 751 760 10.1016/S1473-3099(04)01205-8 15567125

